# Ophthalmic Alterations in the Sturge-Weber Syndrome, Klippel-Trenaunay Syndrome, and the Phakomatosis Pigmentovascularis: An Independent Group of Conditions?

**DOI:** 10.1155/2015/786519

**Published:** 2015-09-16

**Authors:** Solmaz Abdolrahimzadeh, Vittorio Scavella, Lorenzo Felli, Filippo Cruciani, Maria Teresa Contestabile, Santi Maria Recupero

**Affiliations:** ^1^Ophthalmology Unit, DAI Head/Neck, Umberto I Policlinic, University of Rome “Sapienza”, Viale del Policlinico 155, 00161 Rome, Italy; ^2^Ophthalmology Unit, Department of Sense Organs, University of Rome “Sapienza”, Viale del Policlinico 155, 00161 Rome, Italy; ^3^Section of Ophthalmology, Policlinico Militare di Roma, Piazza Celimontana 50, 00184 Roma, Italy; ^4^Ophthalmology Unit, St. Andrea Hospital, NESMOS Department, University of Rome “Sapienza”, Via di Grottarossa 1035-1039, 00189 Rome, Italy

## Abstract

The phakomatoses have been traditionally defined as a group of hereditary diseases with variable expressivity characterized by multisystem tumors with possible malignant transformation. The Sturge-Weber syndrome, Klippel-Trenaunay syndrome, and the phakomatosis pigmentovascularis have the facial port-wine stain in common. Numerous pathophysiogenetic mechanisms have been suggested such as venous dysplasia of the emissary veins in the intracranial circulation, neural crest alterations leading to alterations of autonomic perivascular nerves, mutation of the GNAO gene in the Sturge-Weber syndrome, PIK3CA mutation in malformative/overgrowth syndromes such as the Klippel-Trenaunay syndrome, and the twin-spotting phenomenon in phakomatosis pigmentovascularis. Other features linked to the port-wine stain and typical to all of the three conditions are glaucoma and choroidal alterations. Glaucoma can be due to malformations of the anterior chamber or high episcleral venous pressure and in phakomatosis pigmentovascularis it can also be associated with angle hyperpigmentation. The choroid can be thickened in all diseases. Furthermore, choroidal melanocytosis in the phakomatosis pigmentovascularis can lead to malignant transformation. Although the multiple pathophysiological mechanisms still require clarification, similarities in ophthalmic manifestations make it reasonable to classify these diseases in an independent group.

## 1. Introduction

The Sturge-Weber syndrome (SWS) and Klippel-Trenaunay syndrome (KTS) were included in the phakomatoses together with neurofibromatosis, tuberous sclerosis, and von Hippel-Lindau syndrome in 1937 [1]. In support of this hypothesis, and based on histopathological observations, Hogan and Zimmerman [2] in 1962 suggested that the phakomatoses are multisystem hamartoses regardless of the risk of malignant transformation. Since then many authors have included SWS and KTS in the group of phakomatoses whereas others have defined them as “the odd men out” [3–7]. The facial port-wine stain is a characteristic of the SWS, KTS, and phakomatosis pigmentovascularis (PPV). Furthermore, glaucoma and thickened choroid, linked to the port-wine stain, are recurrent ocular findings in all three conditions. Various pathophysiological mechanisms have been proposed, but the clinical similarities, ophthalmic manifestations in particular, make it reasonable to classify these diseases as an independent group.

## 2. Sturge-Weber Syndrome

The earliest case regarding SWS was reported in 1860 by Schirmer. The patient had bilateral facial nevus as well as unilateral buphthalmos [8]. In 1879, Sturge reported on a case with bilateral facial nevus, vascular deformity, and congenital glaucoma in the right eye and spasms affecting the patient's left side of the body [9]. Then, in the year 1922, the first radiographic evidence of intracranial calcifications was brought forth by Weber [10]. The ophthalmologist van der Hoeve was the first to describe the phakomatoses as a clinical entity of diseases including tuberous sclerosis, neurofibromatosis, and von Hippel-Lindau and Sturge-Weber syndromes [11].

SWS also known as encephalotrigeminal angiomatosis includes naevus flammeus, also known as port-wine stain (PWS), and ipsilateral leptomeningeal angiomatosis as the main features [6]. Estimated frequency is about one in 50,000 live births. This syndrome affects both men and women at a seemingly parallel rate [12].

The pathogenesis of the port-wine stain (PWS) is still not completely understood, but it is linked to progressive ectasia of the superficial cutaneous vascular network [13, 14]. Some authors have suggested that the PWS is related to disorders of neural crest cells [15, 16]; ultrastructural and immunohistochemical studies have demonstrated the absence of perivascular nerves in PWS [14, 17] favouring the hypothesis of an alteration of autonomic nerves surrounding blood vessels which causes deficits of vessel caliber modulation [14, 18]. In the recent years, various authors have proposed that the SWS (and the KTS) should not be classified among other phakomatoses as there is no hereditary pattern or predisposition and the manifestations of both conditions are those of hypertrophy rather than the hyperplasia characteristic to phakomatosis [19], and there is no malignant transformation [11]. In original studies, Parsa elaborated a pathophysiologic mechanism attributing the vascular ectasia in PWS to dysplasia of the emissary veins in the peripheral intracranial circulation resulting in increased retrograde venous pressure within the communicating vessels and the superficial venous plexus of the skin implying that SWS and KTS are products of “acquired venous obstruction rather than neural dysfunction” [20]. Moreover, the author suggested that when venous dysplasia involves the limbs it causes tissue hypertrophy [19, 20]. The presence of combined SWS and KTS has been challenged and it has been advanced that patients diagnosed with KTS who present capillary deformities at a level inferior to the head, in the absence of lymphatic pathologies, are actually afflicted with a variant of SWS [21].

Shirley et al. recently identified a mutation in the GNAO gene, which stimulates the proliferation of cells and inhibits apoptosis by a surge in downstream signaling through the RAS effector pathways [22]. The mutation probably takes place earlier on in SWS with respect to isolated PWS and seems to underlie both of these conditions [22].

Histological evidence has shown that both cerebrovascular and cutaneous lesions correspond to initial localized venous dysplasia during the 4th and 8th week of pregnancy [23]. Some neurologists have suggested that during week 9 the vascular plexus fails to regress in SWS. A lack of ordinary leptomeningeal vessels and blockage due to vascular deformity may lead to hypoxia, ischemia, stasis, and a decline in neuronal metabolism, which is noted particularly when seizures occur [23]. Clinical signs of SWS usually consist of unilateral facial PWS, ipsilateral glaucoma, hemianopia, hemiatrophy, progressive seizures, contralateral hemiparesis, and mental deficiencies [24]. Intracranial lesions appear in the manner of gyriform or “tram-line” calcifications that tend to engage the occipital and parietal lobes, leptomeningeal angiomatosis, neuronal loss, and astrogliosis in brain tissue [25].

Stemming from vascular deformities, which affect the face, eyes, and the leptomeninges, the syndrome's manifestations have been divided depending on vascular deformity distribution.

### 2.1. Cutaneous Signs

**Figure 1:**
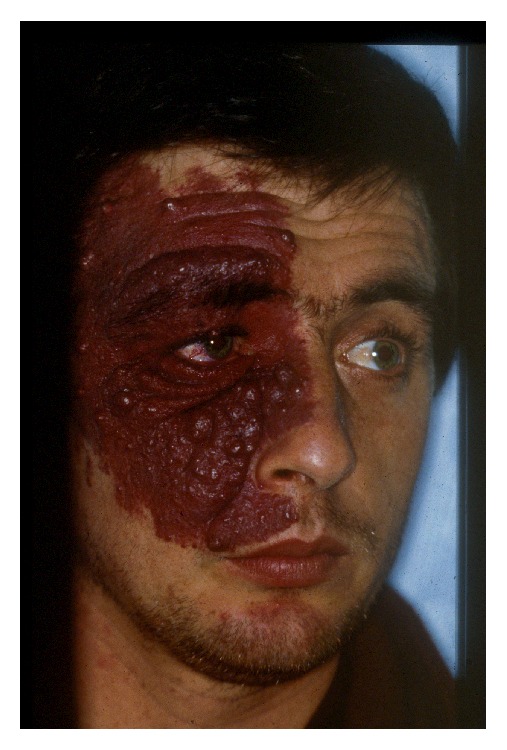
Facial port-wine stain in a patient with Sturge-Weber syndrome.

The most prominent clinical finding of SWS is unilateral facial PWS from birth (Figure 1).

**Figure 2:**
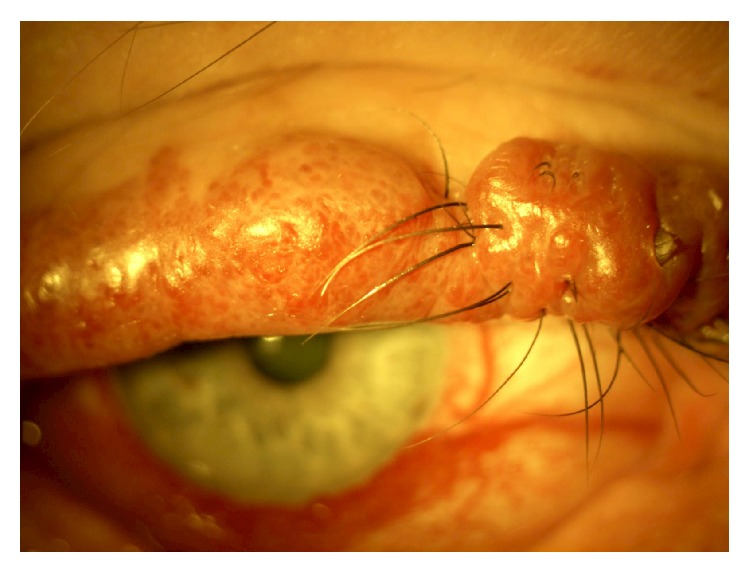
Port-wine stain of the upper lid with nodularity in a patient with Sturge-Weber Syndrome.

It is a well-defined macular lesion initially pink in colour with a smooth surface that, unlike hemangiomas, partially blanches with pressure [26]. The lesion develops proportionally with the child and usually gets darker in color [27]. The skin over the PWS can present nodularity or hypertrophy in about 60% of patients above the age of 50 [28] (Figure 2).

PWSs have been commonly described to affect the first sensory distribution of the trigeminal nerve; however, they can even engage the second and the third distribution areas and may be diffuse and bilateral [29]. Waelchli et al. suggested that facial PWSs appear to trace the face's embryonic vasculature as opposed to the trigeminal nerve. Furthermore, they suggest that the prediction of SWS can be based on facial PWS phenotype where those involving the forehead are associated with seizures, neurodevelopmental abnormalities, atypical brain magnetic resonance imaging (MRI), or glaucoma, suggesting diagnosis of SWS [30].

Histopathologic research has shown comparability between SWS related and isolated cases of nevus flammeus. Principle differences include an increase in dilated thin-walled capillaries and venules mainly situated in the superior segment of the reticular dermis in SWS [31].

### 2.2. Cerebral Findings

Leptomeningeal angiomatosis often affects the parietal and occipital lobes and is characterized by numerous vessels with thin walls, characteristically enlarged and tortuous, within the leptomeninges. These particular vessels are subject to thrombi [32]. A typical sign of SWS is seizure due to microcirculatory disorders and hypoxia. Seizures that occur in patients who have developed the syndrome by 3 years of age tend to take place on the contralateral side to neurocutaneous signs. These seizures affect 70–90% of patients and get worse with time [33, 34]. Infantile spasms and generalized seizures have also been reported [24]. Further consequential neurological signs to leptomeninges angioma include cephalgy, contralateral hemiparesis, stroke-like episodes, hemianopia, and hemiatrophy. Mental retardation also develops in about 50–60% of patients affected by SWS [35].

MRI is superior to computerized tomography in radiologically outlining typical gyriform parietal and occipital calcifications. Intracranial calcifications are present in roughly 90% of younger patients. Infants, however, may present minimal or total absence of calcifications. The examination of choice to evaluate the extent of vascular deformities is MRI with contrast [35].

### 2.3. Ocular Manifestations

**Figure 3:**
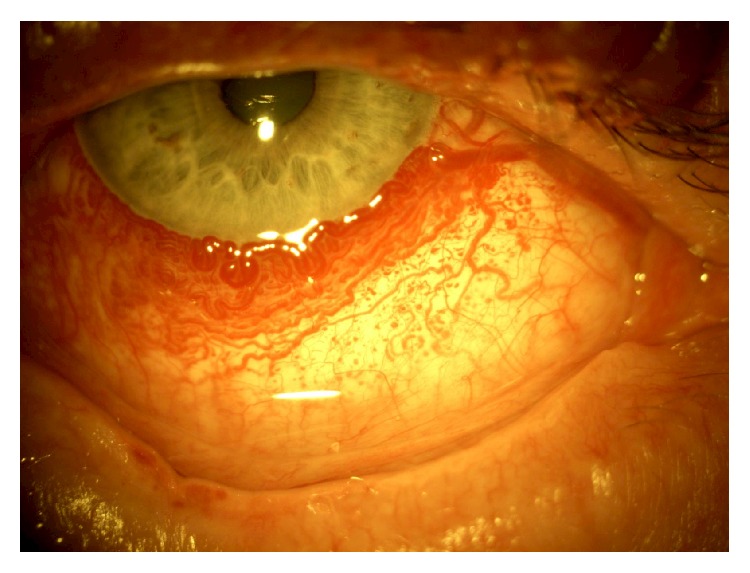
Diffuse conjunctival vascularity in a patient with Sturge-Weber Syndrome.

In as many as 50% of cases the eye is affected. Ocular circulation might be abnormal when skin lesions involve the eyelids. Increased conjunctival vascularity usually produces a pinkish discoloration, which can be diffuse or localized in areas of the bulbar conjunctiva especially in the limbus zone (Figure 3).

Of all SWS related ocular complications, the most common is glaucoma, which affects 50% to 70% of SWS patients [36, 37].

Anterior chamber angle anomalies cause infant glaucoma in roughly 60% of patients while raised episcleral venous pressure triggers glaucoma in 40% of youth and young adult patients [36]. Thus, glaucoma corresponds to two forms depending on the age of development: early-onset (congenital) or later-onset forms. The affected eye presenting glaucoma tends to almost always be ipsilateral to the PWS [37]. The risk of glaucoma is higher when the PWS involves both lids of the eye as opposed to the upper lid only, 72% versus 21%, respectively [36].

There are two major theories on the pathogenesis of glaucoma.

- The first is a mechanical pathogenesis linked to a malformation of the anterior chamber angle with consequent increase of resistance to the outflow of the aqueous humor. This is not necessarily associated with flat anterior iris insertion, which is a feature of the congenital form [37].
- The second theory involves a high episcleral venous pressure, theorized by Weiss in 1973 [38], according to which the arteriovenous shunts are the cause of high episcleral pressure in patients with episcleral hemangioma. This pathophysiological mechanism is supported by reports of the presence of blood within Schlemm's canal [38, 39]. Also, glaucoma is more pronounced in the presence of a correspondingly greater episcleral hemangioma [40].

Maruyama et al. described a patient with SWS who developed angle-closure glaucoma and showed posterior scleritis with edema of the ciliary body, ciliochoroidal effusion, and anterior rotation of the ciliary body, as well as inflammation of the crystalline lens, which caused closure of the chamber angle [41]. Various disorders can cause ciliochoroidal effusion: venous congestion, ocular inflammation, and other factors or drugs associated with traumatic, idiopathic, neoplastic, and systemic diseases [42, 43]. Acute glaucoma arises as the consequence of ciliary body effusion, which moves forward the iridolenticular diaphragm reducing the amplitude of the anterior chamber angle. This can be observed with ultrasound biomicroscopy, which provides images of the anterior segment, chamber angle, and the ciliary body [44].

Light microscopy has shown clusters of vascular formations in the trabecular meshwork near the scleral spur surrounded by large homogeneous extracellular matrix. Electron microscopy showed that the endothelial layer lining of Schlemm's canal was associated with basal lamina. The endothelial cells contained several villi and giant vacuoles, which appeared to be transcellular channels [45].

The choroid is the site of the most important vascular alteration associated with SWS. Hemangiomas of the choroid occur in two specific forms: circumscribed forms typically occur in patients with no other systemic disorders and diffuse forms are seen in SWS [46]. In most cases, an increase of well-formed choroidal vessels gives the fundus a consistent bright red or red-orange color [19]. Choroidal hemangiomas usually remain asymptomatic throughout childhood. However, in adolescence or adulthood, the choroid sometimes becomes markedly thickened [47].

Histological features of choroidal angioma in SWS are different from those of circumscribed choroidal angiomas. In SWS, choroidal vasculature does not show proliferation of vessels, pericytes, or endothelial cells [48].

Degeneration or detachment of the overlying retina is a severe complication [49]. Vision loss can consequentially occur following subretinal hemorrhage and serous retinal detachment. Further complications are photoreceptor alteration, cystoid macular oedema, and serous detachment of the neuroepithelium in the macular area [50, 51].

Diagnosing choroidal angioma is achieved through fundus examination with indirect ophthalmoscopy, by which the difference in color of the fundus between fellow eyes can be distinguished and the extension of choroidal involvement can be determined in some cases. Retinography may also aid in the differentiation in color of fellow eyes. Instrumental examinations, important for the diagnosis of choroidal hemangioma, are ultrasonography, indocyanine-green angiography, and enhanced depth imaging (EDI) spectral domain optical coherence tomography (SD-OCT). Ultrasonography confirms the presence of choroidal lesions together with their extension, echogenicity, and characteristics. Indocyanine-green angiography highlights the extension, intralesional vascularity, and possible arteriovenous communications of the choroidal lesions. Finally, EDI SD-OCT allows for extensive assessment of choroidal and any related retinal alterations at the posterior pole [47]. Furthermore, choroidal thickness and morphology and evaluation of the caliber of dilated choroidal vessels can be evaluated with OCT [52].

### 2.4. Diagnostic Criteria

The main characteristics of SWS include ipsilateral leptomeningeal angiomatosis in the parietal-occipital lobe, unilateral facial PWS, and congenital glaucoma. It is worth noting that these signs are usually manifested only in part.

SWS is divided into four categories:

1. classic SWS: leptomeningeal and facial angiomas; glaucoma possible;
2. PWS without evidence of cerebral involvement;
3. isolated leptomeningeal angioma;
4. classic form with other systemic associations, such as tuberous sclerosis.

### 2.5. Treatment of Port-Wine Stains

For cases of facial PWSs, laser treatment ought to be initiated immediately for optimal results. Success of laser treatment is dependent on the location of the deformity. Lesions affecting the central forehead yield better results as opposed to central facial lesions. Unfortunately, PWSs that are left untreated through the years have a tendency to thicken and get darker and, in some cases, nodularity can even develop [33].

A PWS can be eliminated with deep photocoagulation and debulking surgery if there are hypertrophic alterations [19]. However, according to Parsa, this treatment may create a reduction of cerebral venous outflow through collateral vessels of the PWS, thus, potentially worsening cerebral and ocular blood flow anomalies which could lead to cerebral venous deterioration, increase in intraocular pressure, choroidal vessel dilatation, and detachment of the retina due to exudation. According to this author, careful superficial laser treatment would not affect the deep blood circulation or alter the collateral circulation, avoiding these complications [19].

### 2.6. Treatment of Glaucoma

In SWS related glaucoma, standard management includes lifelong medical treatment as well as surgery. Controlling intraocular pressure (IOP) for optic nerve damage prevention remains the main objective. According to Yang et al. [53] uveoscleral outflow is due to elevated episcleral venous pressure and causes disruption of natural aqueous drainage processes. In a study by Basler and Sowka [40], IOP in about 50% of patients appeared to be successfully controlled by latanoprost in SWS glaucoma. Topical therapy with beta-blockers and carbonic anhydrase inhibitors in conditions of reduced outflow is effective where buphthalmos is not present [54].

However, topical medication in itself is not sufficient in managing SWS-associated glaucoma. Hence, surgery frequently supplements superior long-term management of IOP. The most appropriate surgical approaches in children under four years of age are trabeculotomy and goniotomy although the long-term results have been disappointing [40]. Second-line treatments are filtering procedures: trabeculectomy, posterior lip sclerectomy [39], and trabeculotomy-trabeculectomy [55, 56]. Trabeculotomy eases outflow by overcoming the anterior chamber angle abnormalities, whereas trabeculectomy bypasses the episcleral venous system by creating an alternative outflow. Topical medications are commonly the first-line therapy for patients who develop glaucoma in later stages of life [40]. Filtering procedures may result in more severe problems including bleeding, expulsive choroidal hemorrhage, and a prolonged flat anterior chamber [57, 58].

van Emelen et al. [59] demonstrated the effectiveness of cryocoagulation in combination with topical treatment in a case series of SWS patients with buphthalmos. The efficacy of cyclocryotherapy in addition to trabeculectomy has also been evaluated; however, the complications of extensive cyclocryotherapy are phthisis bulbi and chronic hypotony [60]. The choice of treatment parameters is critical in order to prevent the onset of ocular hypotony. Cyclodestructive procedures have a greater hypotensive effect.

The use of Ahmed valve implantation, which allows the outflow of aqueous with increased performance on the long term, allows us to avoid trabeculectomy and its high risk of intraoperative hemorrhage and suprachoroidal effusion [61]. The Molteno tube has also been used in a small case series in children suffering from SWS. Though the outcomes were not favourable and there was as an elevated complication rate [62].

### 2.7. Treatment of Choroidal and Retinal Alterations

The principal motive for visual deterioration in patients with SWS-associated choroidal hemangioma is the accumulation of macular subretinal fluid. Treatment is aimed primarily at reducing tumor leakage and also to the destruction of the tumor itself [63]. The most effective and commonly utilized treatment is photocoagulation. Confluent photocoagulation causes destruction of the tumor resulting in reduced leakage [64]. Intense photocoagulative treatment is associated with many complications; thus, some authors have opted for lighter grid treatment [65, 66]. Nonetheless, subretinal fluid recurrence is more frequent with this less intense treatment.

Photodynamic therapy (PDT) is used to lessen subretinal macular fluid. Theoretically, PDT triggers atrophy of the hemangioma vessels and reduces leakage. PDT has been efficient in treating cases of choroidal hemangioma with noteworthy reduction of leakage, even in tumors adjacent to the fovea [66].

External beam radiotherapy (EBR) is used in treating diffuse choroidal hemangiomas associated with exudative retinal detachment [67, 68]. The results are obtained months after the first application and relapse is frequent; thus repeated applications can cause radiation retinopathy, neuropathy, and cataract [67]. In 1960, MacLean and Maumenee recounted the first attempt using brachytherapy coupled with radon seeds to treat circumscribed choroidal hemangiomas [69]. Brachytherapy with Cobalt-60 [70] and Ruthenium-106 [71, 72] showed satisfactory results in reducing exudative retinal detachment in choroidal hemangiomas. Retinal detachment is a rare complication that is surgically manageable, but in one case of exudative detachment intravitreal antivascular endothelial growth factor therapy with pegaptanib showed good results [73].

## 3. Klippel-Trenaunay Syndrome

The Klippel-Trenaunay syndrome, which was initially illustrated by Klippel and Trenaunay in 1900 [74], is a rare multisystem disorder, which has an incidence of about 1 : 100,000 with no predilection for gender, race, or geographical area and most cases are sporadic [75, 76]. The characteristic triads of congenital anomalies are PWS, varicose veins, and bony and soft-tissue hypertrophy. When this clinical picture is associated with arteriovenous shunting the condition has also been called the Parks-Weber syndrome [77].

In 1960, Pietruschka suggested that the SWS and KTS are one and the same disease [78]. Sharma et al. suggested that the syndromes are closely related and should be named neurocutaneous angioma since there may be different expressions of a disease with a sole pathophysiological mechanism [79]. Both conditions share the presence of the PWS. Parsa suggested that the alterations associated with the PWS in KTS arise when venous dysplasia is in the lower extremities (or below the heart level) where venous drainage is poor causing tissue pressure elevation and cellular hypertrophy [19]. Furthermore, lymphatics and veins share a common embryologic origin; thus, lymphatic dysfunction and malformations can occur in the KTS [19]. Kihiczak et al. proposed that KTS may result from vascular and tissue overgrowth due to a specific pathogenic gene [80]. A familial or paradominant inheritance pattern for KTS and a single gene translocation etiology has also been suggested by some authors [81, 82]. Recently, hypermorphic somatic phosphatidylinositol-4,5-bisphosphate 3-kinase and catalytic subunit alpha (PIK3CA) mutations have been found in various patients with malformative/overgrowth syndromes [83]. It has been postulated that the mechanism of malformation and overgrowth during embryogenesis is due to the alteration of multiple signalling pathways including the insulin-like growth factor, vascular endothelial growth factor, and fibroblast growth factor pathways [84].

**Figure 4:**
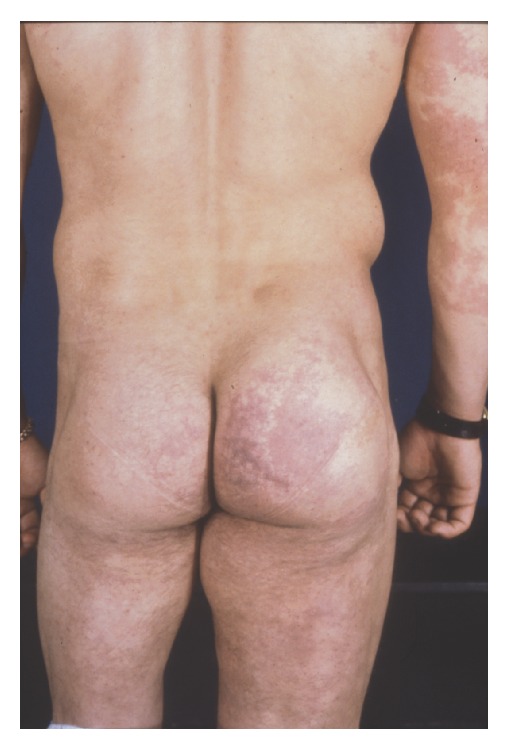
Soft-tissue and bony hypertrophy of the lower limb in a patient with Klippel-Trenaunay Syndrome from [7].

The condition is commonly seen at birth or early childhood and appears with a PWS, which is present in 98% of patients [85, 86]. Varicose veins are seen more frequently during adolescence and can involve both the deep and superficial venous plexuses; these can be complicated by lymphedema, thrombophlebitis, and ulcers [87]. The cutaneous alteration can be limited to the skin or involve organs such as the colon, liver, spleen, or bladder and can lead to internal hemorrhage [80]. Hypertrophy of soft tissues and bone is more frequently for the lower limbs but any part of the body can be affected with variable extension, which can even be limited to only the fingers or toes or may be severe with massive limb overgrowth [87, 88]. Mental retardation can be encountered especially when patients present hemangiomas of the face and head. Diagnosis of the disease is when at least two signs among the following are present: PWS, varicose veins, soft-tissue, and/or or bony hypertrophy but 63% of diagnosed patients present all three symptoms [89] (Figure 4).

### 3.1. Ophthalmic Features

**Figure 5:**
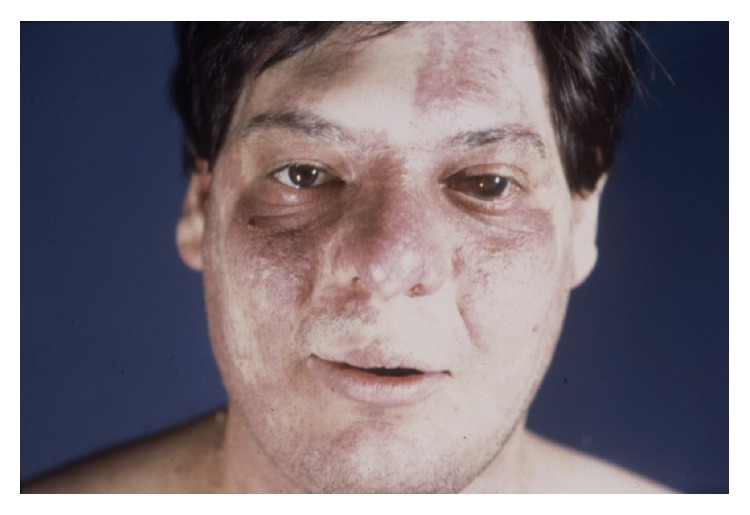
Bilateral facial port-wine stain and glaucoma of the left eye in a patient with Klippel-Trenaunay Syndrome from [7].

The most common ophthalmological alterations encountered in the KTS are choroidal hemangiomas similar to those described for the SWS [90]. Glaucoma, also frequently observed, has been associated with anterior chamber malformation [91] or due to raised episcleral venous pressure as in the SWS [38, 39] (Figure 5).

Other ophthalmic alterations have been reported in case studies and consist in: conjunctival telangiectasia, orbital varix, strabismus, oculosympathetic palsy, Marcus-Gunn pupil, iris coloboma and heterocromia, cataracts, persistent fetal vasculature, chiasmal and bilateral optic nerve gliomas, drusen of the optic disk, acquired myelination of the retinal nerve fiber layer, and retinal dysplasia with astrocytic proliferation of the nerve [91–97]. Retinal varicosities have been described and dilated retinal veins have been demonstrated with fluorescein angiography [98].

Very little information is available in the literature regarding the treatment of the ophthalmic manifestations of the KTS but this overlaps with the management of glaucoma and choroidal alterations described for the SWS.

## 4. Phakomatosis Pigmentovascularis

Phakomatosis pigmentovascularis was first described by Ota et al. in 1947 [99]. Toda in 1966 and Hasegawa and Yashara in 1979 described further variants [100, 101]. It is a neurocutaneous condition where a naevus flammeus is found in association with pigmentary nevi involving the eye (ocular melanocytosis) or the face and body (oculodermal melanocytosis) [99]. PPV has been described prevalently in Asian patients [99, 101, 102] although rare cases in other races have been described [103]. The pigmentary nevi can be nevus spilus or Mongolian spots [99, 104, 105]. Nevus spili are brown, polymorphous spots secondary to epidermis basal layer hyperpigmentation without involvement of the underlying derma [7]. Mongolian spots are formed by an accumulation of melanocytes, which are filled with melanin in the mid and deep derma. They are patchy areas of variable morphology with a colour that can range from bluish to brownish [7]. Ocular melanocytosis or nevo di Ota is when the skin along the ophthalmic, maxillary, and more rarely the mandibular branch of the trigeminal nerve is involved and if the hyperpigmentation only involves the eye it is termed melanosis oculi. 30% of patients have hyperpigmentation of ocular structures [106]. The nevus flammeus or PWS is similar to that occurring in the SWS and KTS [13]. Indeed, PPV has been reported most frequently with SWS and KTS syndromes or SWS alone [103, 107–111].

Neural crest alterations have been long held as a principal cause of many of the manifestations of phakomatoses such as neurofibromatosis [112]. Indeed, research in neurofibromatosis type 1 has shown retinal microvascular alterations [113] even overlying choroidal nodules typical of the diseases [114] suggesting the hypothesis that choroidal and retinal thinning in NF1 could be caused by altered innervation of perivascular vessels due to neural crest cell abnormalities [114]. Furthermore, in PPV oculodermal melanocytosis, Mongolian spots and dermal melanocytosis are derived from aberrant migration of neural crest cells [99, 104]. This theory is based on altered vasomotor regulation, which leads to the formation of phakomas and the naevus flammeus [104, 115]. It is interesting that the association of PPV with neurofibromatosis and Lisch nodules has been described on several occasions [116, 117]. Progress in genetic research has led us to consider that the association of melanocytosis and PWS occurs due to the twin-spotting phenomenon. It is believed that this was produced by somatic recombination where there are two genetically distinct cell clones within an area of cells, which are normal. Therefore, there is a mosaic distribution of alterations and the condition is sporadic without familial transmission [118].

PPV has been classified by various authors [99–101, 104, 106]. Happle divided this group of conditions into 3 distinct types: phakomatosis cesioflammea, phakomatosis spilorosea, and phakomatosis cesiomarmorata [119]. The most frequent type is cesioflammea in 77% of cases followed by spilorosea in 13% of cases and rarely cesiomarmorata; finally, 8% remain unclassified [120]. Phakomatosis cesioflammea features dermal melanocytosis (blue spot) and nevus flammeus, which can be associated with nevus anemicus, focal alopecia, glaucoma, ungueal hypoplasia, and limb asymmetry [118, 121]. Phakomatosis spilorosea involves nevus spilus (speckled freckled nevus) and a telangiectatic nevus with a light pink color (lighter with respect to the naevus flammeus). These can be combined with other symptoms such as hemiparesis, seizures, lymphedema, and limb asymmetry [118]. Finally, the rare phakomatosis cesiomarmorata is the combination of nevus caesius (Mongolion spot or blue-gray nevus) and cutis marmorata telangiectatica congenita, which can be associated with blue sclera, leg hyperplasia, and asymmetric cerebral hemispheres [118].

Ocular manifestations in PPV encompass hyperpigmentation of the conjunctiva, sclera, episclera, iris, trabecular meshwork, and the choroid [118, 122]. Furthermore, melanocytosis of the corneal stroma and pigmentary deposits on the lens have been described [106, 123]. Iris mammillations are also found in association with melanosis oculi [124–126]. These are protuberances, which can have a smooth, villiform, conical, or stellate appearance that can partially or completely cover the iris on its surface giving it a dark and smooth appearance [126]. At times, these mammillations can be confused with Lisch nodules typical of neurofibromatosis type 1, but Lisch nodules have an irregular distribution on the iris face and are variable in number and dimensions [126, 127]. Ten percent of patients with oculodermal melanocytosis present glaucoma and the mechanism can be due to angle hyperpigmentation or the increase in aqueous outflow resistance due to melanocytes [128]. Congenital or developmental glaucoma [106, 123, 129] has been suggested due to abnormal neural crest development, which leads to anomalous anterior chamber angle [122]. In nine cases described by Teekhasaenee and Ritch, all patients, with both melanocytosis involvement and episcleral vascular involvement for 360 degrees, developed congenital glaucoma [122]. The authors concluded that there is high risk for congenital glaucoma when there is extensive ocular involvement. Partial involvement of the globe, however, predisposes to high IOP, which can develop later in life. Naevus flammeus has been reported to be more strongly associated with glaucoma than oculodermal melanocytosis and the mechanisms involved are those described in the section regarding glaucoma in the SWS.

Patients with oculodermal melanocytosis have a greater risk to develop melanoma of the uvea, 1 in 400 white patients with oculodermal melanocytosis with respect to 6 per million for the general population [130]. In PPV, melanocytosis of the fundus has frequently been described and choroidal melanoma can develop as shown in 3 of 6 patients described by Shields et al. in 2011 and 5 patients by Tran and Zagrafos [118, 131]. Ocular melanocytosis is not always readily visible on external examination and careful fundus examination of patients with PPV, in order to monitor for uveal melanoma, is strongly advised by some authors [70, 118]. Ultrasound biomicroscopy is also advised for evaluation of the ciliary body [132].

There is very little information in the available literature on the management of ophthalmic conditions in the phakomatosis pigmentovascularis. Filtering surgery in association with antimetabolites has been reported [118]. However, similar to reports in the SWS, there is a high risk of suprachoroidal hemorrhage during surgery.

## 5. Conclusions

Our present knowledge on the multiple pathophysiological mechanisms involved in SWS, KTS, and the PPV does not allow us to determine whether these rare conditions should be included among the phakomatoses. It would seem reasonable to embody these diseases in a group of their own as there may be diverse manifestations of the same spectrum, which would explain the similarities in some clinical aspects. The complex pathophysiology and the role of the neural crest, venous dysplasia, and novel mutations require further clarification.
